# Cyst formation in the subchondral bone following cartilage repair

**DOI:** 10.1002/ctm2.248

**Published:** 2020-12-13

**Authors:** Liang Gao, Magali Cucchiarini, Henning Madry

**Affiliations:** ^1^ Center of Experimental Orthopaedics Saarland University Medical Center and Saarland University Homburg Germany

**Keywords:** bone cyst, cartilage repair, osteochondral unit, subchondral bone

## Abstract

Subchondral bone cysts represent an early postoperative sign associated with many articular cartilage repair procedures. They may be defined as an abnormal cavity within the subchondral bone in close proximity of a treated cartilage defect with a possible communication to the joint cavity in the absence of osteoarthritis. Two synergistic mechanisms of subchondral cyst formation, the theory of internal upregulation of local proinflammatory factors, and the external hydraulic theory, are proposed to explain their occurrence. This review describes subchondral bone cysts in the context of articular cartilage repair to improve investigations of these pathological changes. It summarizes their epidemiology in both preclinical and clinical settings with a focus on individual cartilage repair procedures, examines an algorithm for subchondral bone analysis, elaborates on the underlying mechanism of subchondral cyst formation, and condenses the clinical implications and perspectives on subchondral bone cyst formation in cartilage repair.

## Graphical abstract

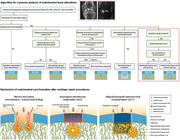

## 1 BACKGROUND

Articular cartilage, the resilient and flexible connective tissue covering the articulating surfaces of joints, has a limited regenerative capacity.^1^ Regeneration of chondral (limited to the cartilage) and osteochondral defects (extending into the subchondral bone) refers to an identical reconstruction of the original osteochondral unit. However, in adults, only different degrees of repair occur, all resulting in a structurally and functionally inferior (osteo)chondral repair tissue.^2^, ^3^, ^4^, ^5^ Present major reconstructive surgical interventions for focal cartilage defects include marrow stimulation, osteochondral allograft or autograft transplantation (OCT), and autologous chondrocyte implantation (ACI).^2^, ^3^, ^4^, ^5^ Indications for these approaches are symptomatic cartilage defects with unsatisfactory outcomes after sufficient conservative therapies, aiming at preventing secondary degenerative processes.^6^, ^7^, ^8^, ^9^ To identify an appropriate surgical modality, the following critical issues need to be considered: etiology of the defect, patient's age, body mass index, physical activity level and expectations, mechanical axis, possible comorbidities, and defect characteristics such as size, number, and location.^10^, ^11^, ^12^, ^13^, ^14^ If correctly indicated, such cartilage repair techniques yield largely satisfactory outcomes.^15^ Clinical outcomes of cartilage repair are usually assessed using different joint function scores, patient reported outcome measures, and structural evaluations such as the nondestructive MRI^16^ and Arthro‐CT^17^ imaging. Rarely, macroscopic or even microscopic evaluations (based on biopsies) of the repair tissue during second‐look arthroscopy are performed.^18^, ^19^, ^20^, ^21^, ^22^, ^23^, ^24^, ^25^, ^26^, ^27^, ^28^, ^29^, ^30^, ^31^

Currently, a focus of research has been expanded from exclusively regarding the cartilaginous repair tissue to a more complex view including also postoperative structural alterations of the subchondral bone, as they have emerged as a source of considerable clinical problems and thus are being recognized as additional factors influencing long‐time clinical outcomes of various cartilage repair procedures.^32^ Among those, cyst formation in the subchondral bone has been recently described and identified as an important postoperative pathology that may affect the articular cartilage repair.

Bone cysts in general are one of the most widely reported bone changes. From a clinical perspective, they are often causing pain and may reduce the range of motion and of the overall joint function. Bone cysts might result from external (trauma) or multiple internal etiologies such as osteoarthritis (OA), the major degenerative joint disease,^33^, ^34^, ^35^, ^36^ rheumatoid arthritis (RA),^37^, ^38^ intraosseous ganglia,^39^, ^40^, ^41^ aneurysmal bone cysts (ABC),^42^, ^43^, ^44^, ^45^ and articular cartilage defects.^46^, ^47^, ^48^ As such bone cysts alter the structural support for weightbearing, they potentially undermine the biomechanics of the joint, inducing degeneration of the overlying articular cartilage, subchondral collapse, and fracture, all leading to a possible extension into formerly unaffected areas in the form of OA. In the worst case, such changes may progress and necessitate a total knee arthroplasty.^15^

Nevertheless, a clear definition and comprehensive analysis focusing on subchondral bone cyst formation in the context of focal, non‐OA articular cartilage defects and their repair are largely lacking. The aims of this review are to present an algorithm for analysis and a definition of subchondral bone cysts following cartilage repair, discuss mechanism of their formation, and provide a comprehensive overview of such cysts reported in preclinical and clinical studies of cartilage repair.

**Highlights**

- Subchondral bone cysts commonly occur adjacent to a treated focal cartilage defect and are possibly connected to the joint cavity.
- A radiographic‐based algorithm allows for a detailed analysis of postoperative subchondral bone cysts and other alterations of the subchondral bone.
- Formation of subchondral bone cysts might result from synergistic effects of both external and internal contributors.

## 2 A SYSTEMATIC ANALYTIC ALGORITHM FOR CYST FORMATION IN THE SUBCHONDRAL BONE FOLLOWING CARTILAGE REPAIR

**FIGURE 1:**
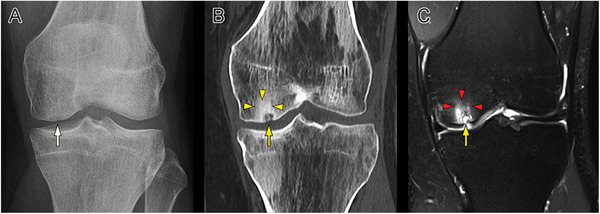
Radiographic images of a 30‐year‐old male patient with osteochondritis dissecans (OCD) (A) at the left medial femoral condyle initially treated with the subchondral drilling and subsequent symptomatic subchondral bone cyst formation (B, C) at 60 months postoperatively. The white (A), yellow (B), and red arrows (C) indicate the subchondral bone cyst. The yellow arrowheads (B) designate the area of the OCD lesion surrounding the subchondral bone cyst in the CT image. The red arrowheads (C) denote the high signal intensity of the diffuse bone marrow edema (BME) around the cyst in the T2‐weighted MRI image

**FIGURE 2:**
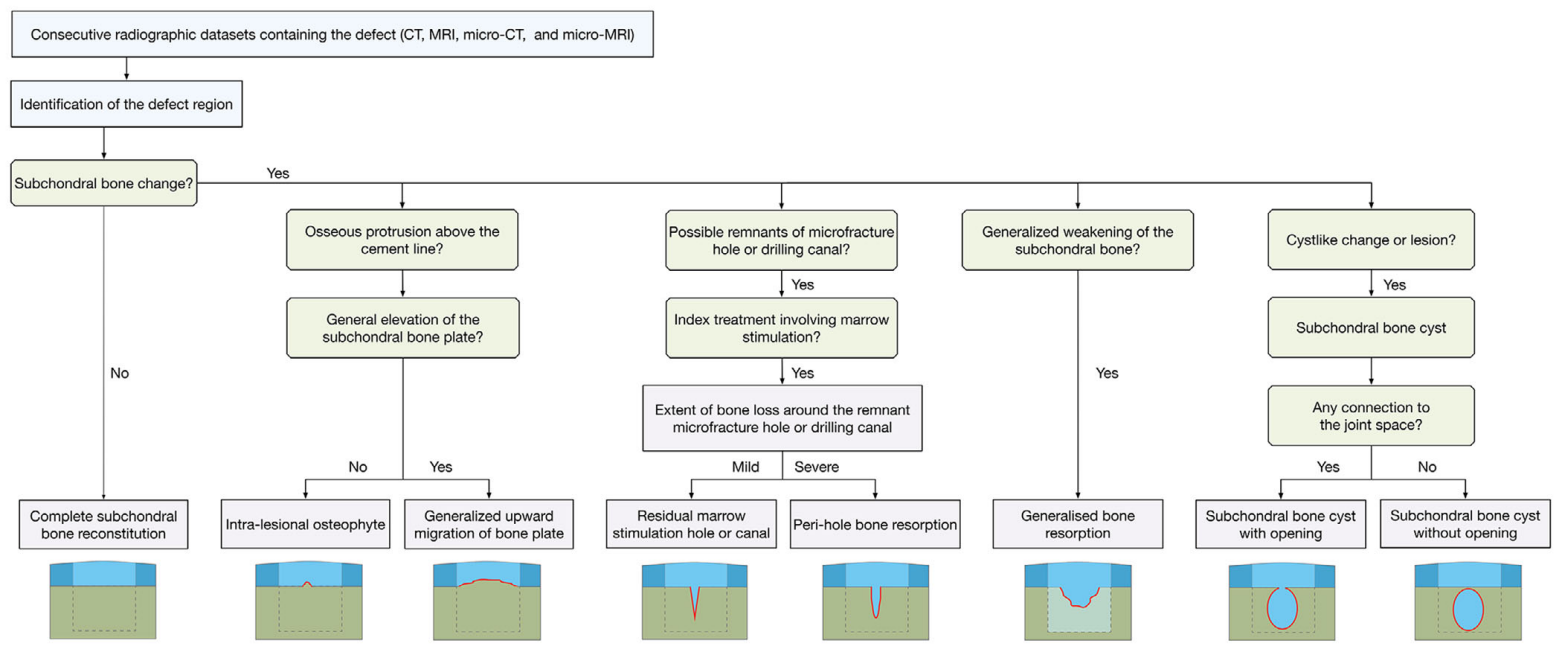
Adapted algorithm for a precise analysis of subchondral bone alterations in translational models and in patients.^52^ The bottom schematics show each pattern of subchondral bone changes with articular cartilage and subchondral bone denoted in dark blue and dark green, respectively. The cartilaginous repair tissue and subchondral bone underlying the defect are depicted in light blue and with dashed border, respectively. The margin of the subchondral bone changes is outlined with red lines. A diffuse bone weakness (light green) is only seen in the generalized bone resorption

In the field of clinical knee OA, algorithms to predict structural progression without specifically addressing subchondral bone cysts support the general concept of subchondral bone evaluations, for example, by quantifying periarticular bone mineral density.^49^, ^50^ Previous analyses of subchondral bone changes in the context of cartilage repair exposed variable patterns, including the formation of subchondral bone cysts (Figure 1), intralesional osteophytes, generalized upward migration of the subchondral bone plate, and the presence of residual marrow stimulation hole(s), together with peri‐hole or generalized bone resorption (Table 1).^51^, ^52^ With a view of systematically exploring each of these morphologic changes in both preclinical^46^, ^47^, ^53^, ^54^, ^55^, ^56^, ^57^ and clinical^25^, ^58^, ^59^, ^60^, ^61^, ^62^, ^63^, ^64^, ^65^, ^66^ settings, an adjustable algorithm has been recently proposed to radiographically analyze them (Figure 2).^52^ In this algorithm, the projected tidemark and cement line serve as topographical landmarks. In the special case of microfracture treatment for cartilage repair, the algorithm utilizes the diameter of the microfracture awl as a constant reference and the dimension of bone void relative to the original microfracture hole as a quantitative standard. The algorithm has been validated and proved to be reliable and reproducible to analyzes datasets from preclinical models of articular cartilage repair, allowing for a precise distinction between each category of subchondral bone changes.^52^ It thus may serve as a useful tool to analyze postoperative subchondral bone cysts and other alterations in an objective and reproducible manner.

**TABLE 1:** Definitions of subchondral bone alterations.^51,52^

Type	Definition
Complete reconstitution	Completely restored subchondral bone underlying the treated defect
Upward migration of subchondral bone plate	Osteochondral junction broadly expanding above its original level, thus subchondral bone plate elevating into cartilaginous repair tissue
Intralesional osteophyte	Focal, newly‐formed bone located apical to its original cement line and projected into cartilaginous repair tissue layer
Generalized upward migration of the subchondral bone plate	Universal expansion of the osteochondral junction above its original level into the cartilaginous repair tissue
Residual marrow stimulation hole	Residual holes or canals originating from marrow stimulation procedures with visible border and opening towards the joint space
Peri‐hole bone resorption	Intermediate bone resorption surrounding the marrow stimulation hole or canal with a possible large opening towards the joint space (may lead to large defects when marrow stimulation holes merge)
Generalized subchondral bone resorption	Generalized weakening of the subchondral bone below the cartilage defect without cyst formation
Subchondral bone cyst	Isolated round or irregular shaped cavity within the subchondral bone with or without connection with the joint space encased by subchondral bone sclerosis

## 3 DEFINITION OF A SUBCHONDRAL BONE CYST IN THE CLINICAL CONTEXT OF CARTILAGE REPAIR

Bone cysts may be categorized according to different pathophysiologies.^67^, ^68^ Osteoarthritic cysts commonly occur in large or small joints with advanced OA,^33^, ^34^, ^35^, ^36^ and are often present within regions of maximal joint space narrowing without or with remaining connections to the joint and thus the synovial fluid. The cysts usually appear within the subchondral bone region, are of spherical or ellipsoid shape, and are associated to other subchondral bone alterations and articular cartilage degeneration.^69^ Sanal et al described them to be located in the subchondral bone below degenerated articular cartilage, lacking a synovial lining.^70^ Associations between subchondral bone cysts and pain along with OA progression have been described especially well in the knee.^34^, ^71^, ^72^ Cyst formation without OA is possible, albeit infrequent.^73^ Such cysts may be present in late RA,^37^, ^38^ pigmented villonodular synovitis (PVNA),^74^ where the invasive inflammatory granulation tissue replaces the subchondral bone. Intraosseous ganglia are benign nonneoplastic intramedullary cysts without signs of OA.^39^ These cysts are usually located in the epiphysis and contain myxomatous fibrous tissue and viscous mucous fluid.^40^ Chondroblastoma is a rare benign tumor, typically leading to a cystic lesion in the epiphyses of long bones.^75^ ABCs represent a different and distinct entity because of their destructive and expansible nature.^43^ They are characterized by a proliferation of connective tissue within blood‐filled cavities.^42^ Sometimes they are accompanied by potentially benign lesions such as chondroblastoma or giant cell tumors.^44^ Polycystic lipomembranous osteodysplasia with sclerosing leukoencephalopathy, also termed Nasu‐Hakola disease, refers to a rare combination of bilateral lytic lesions within the bones of extremities and presenile dementia.^76^

A recent consensus statement from the Society of Skeletal Radiology Subchondral Bone Nomenclature Committee proposed a nomenclature of nonneoplastic conditions involving the subchondral bone and recommended to report the radiological and magnetic resonance imaging (MRI) characteristics of subchondral cyst‐like nonneoplastic conditions with the term "cystlike changes’’ or "cystlike lesion’’ irrespective of their diverse pathologies.^77^ Schajowicz et al used the term “juxta‐articular bone cyst (intra‐osseous ganglion),” for a “benign cystic and often multiloculated lesion made up of fibrous tissue, with extensive mucoid changes, located in the subchondral bone adjacent to a joint."^78^ In the context of joint injury, subchondral bone cysts have been similarly defined by Ziino and Safran as benign cystic and often multiloculated lesions consisting of fibrous tissue located in the subchondral bone adjacent to a joint.^73^ As such subchondral bone cysts are lacking a lining of synovium, the term “synovial cyst” is incorrect.^78^ However, a consensus definition of a subchondral bone cyst in the context of articular cartilage repair has yet to be established.^79^

We propose to define a subchondral bone cyst associated with cartilage repair as an abnormal cavity within the subchondral bone in close proximity of a (treated) cartilage defect with a possible communication to the joint cavity, in the absence of OA. The cyst contains mixed osteo‐chondral‐fibrous tissue with a varying degree of bone remodeling and is often encased with sclerotic subchondral bone. It can be visualized as a pathologic region with well‐defined areas of a fluid signal on MRI corresponding to distinct areas of lucency with a sclerotic rim visible on radiographic or computed tomography (CT) images reflective of the reactive wall around the cyst. Subchondral bone cysts associated with cartilage repair procedures are distinctly different from the many other forms of bone cysts as described above. Compared to OA cysts, their natural history is dissimilar as they are located below a cartilaginous repair tissue, and OA represents a major contraindication for many cartilage repair procedures. Because of the absence of an invasive inflammatory granulation tissue, they are also distinctive from RA‐ and PVNS‐associated cysts. In contract to ABCs, they lack a lytic nature.

## 4 MECHANISMS OF SUBCHONDRAL BONE CYST FORMATION

**FIGURE 3:**
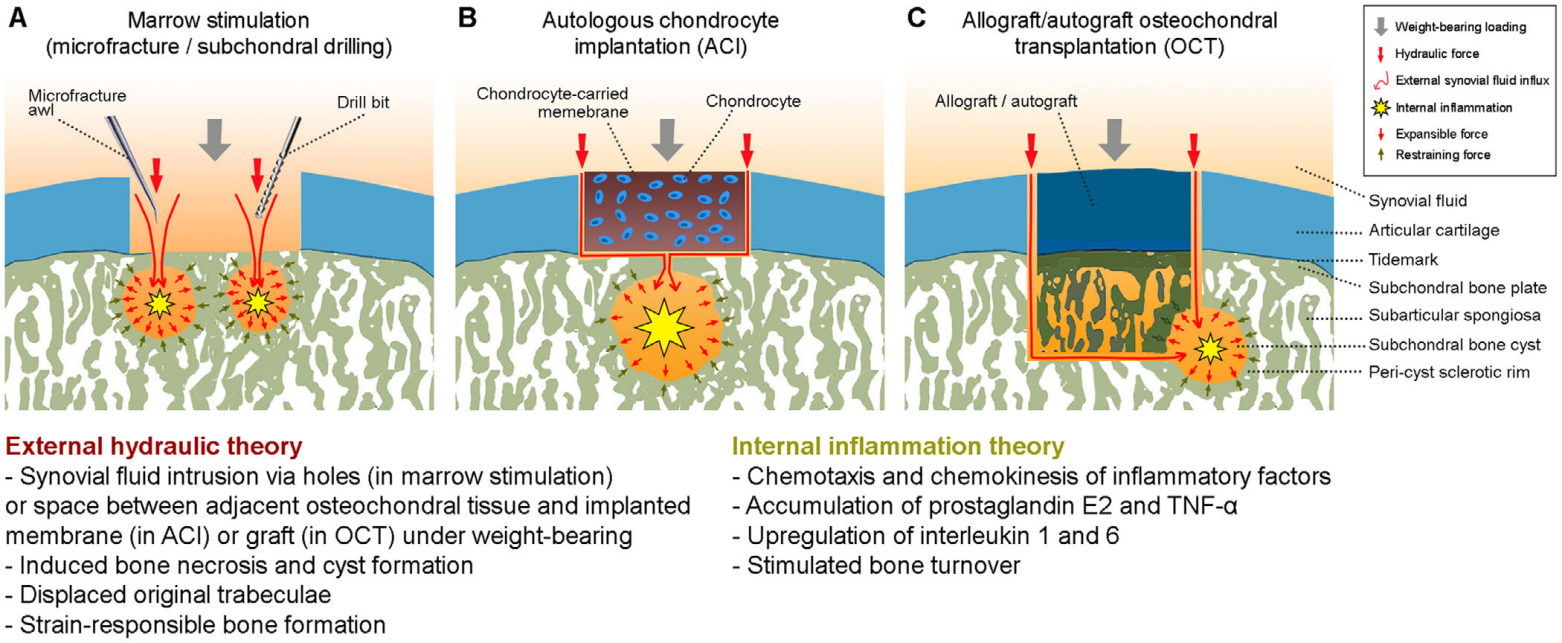
Schematic of the synergistic mechanism of external hydraulic intrusion and internal inflammatory for the subchondral bone cyst formation following articular cartilage repair procedures. The morphological change of the cyst is determined by the equilibrium status between the expansible force due to the synergistic drives and the restraining force from the peri‐cyst sclerotic rim at the cyst‐bone interface. The external hydraulic theory features an intrusion of synovial fluid into the subchondral bone through the canals generated by marrow stimulation techniques (A) or the canals that are possibly opened as a result from the surgically debrided subchondral bone plate in autologous chondrocyte implantation (B) or in a possible gap between the osteochondral unit of the graft and host in allograft/autograft transplantation (C) during the postoperative phase. The pathophysiological characteristics mainly include subchondral bone necrosis, peri‐cyst sclerotic rim formation, displaced original trabeculae and strain‐responsible formation of new bone. The internal inflammatory theory involves mechanisms such as chemotaxis and chemokinesis of inflammatory factors (e.g. PEG2, TNF‐α, IL1, and IL6) as well as bone turnover stimulated by bone necrosis

Understanding the mechanism and pathogenesis of diseases is crucial to identify possible therapeutic targets.^80^ The underlying mechanisms of subchondral cyst formation in the specific context of cartilage repair, albeit of utmost importance, are not yet well understood.^81^, ^82^, ^83^, ^84^, ^85^ Accumulative evidences suggest the synergistic effect of two processes that have been proposed as mechanisms of cyst formation in OA and RA, termed the external hydraulic theory and the internal inflammatory theory (Figure 3).^86^, ^87^

### 4.1 External hydraulic theory

The theory of “blow out" of synovial fluid into the subchondral bone as propounded by Freund^88^ and Landells^89^ requires that a defect in the articular cartilage exists. Prager et al identified a communication of bone cysts with the articular cavity in the form of channels using tomography in 57% of examined cases, although such communication may not always be identifiable.^90^ Such channels might escape their detection using conventional X‐rays or may have become eliminated through bone remodeling.^91^ The external hydraulic theory was supported by work of Ray et al, who demonstrated subchondral bone cyst formation below untreated osteochondral defects at the medial femoral condyles in horses at 24 weeks postoperatively.^92^ The synovial fluid pressure at the exposed subchondral bone generated during the spontaneous postoperative weight‐bearing might be sufficient to induce subchondral bone necrosis and cysts in a step‐wise manner.^89^, ^93^ Besides a physical effect, the synovial fluid itself might contain acellular and cellular elements that may interfere with the subchondral bone. An inhibitory effect of synovial fluid on the tendon healing of a bone tunnel in the context of ligament reconstruction of the knee has been suggested based on preclinical data.^94^, ^95^, ^96^ A short‐term low‐grade synovial inflammation may possibly be induced by the invasive nature of the cartilage repair procedure, shifting the composition of the synovial fluid into a more catabolic and pro‐inflammatory direction. For OA or RA, the deleterious effects of such synovial fluid are well known.^97^, ^98^, ^99^, ^100^ However, a focal cartilage defect represents a comparably less inflammatory and largely nondegenerative condition, a setting in which the aforementioned effects have not yet been investigated. Moreover, a peripheral rim of sclerotic tissue around the cyst is generated during the displacement of the original trabeculae, reflecting a strain‐responding constitution of new bone.^86^

The use of reconstructive surgical repair procedures for cartilage defects deserves special attention in this context. Here, subchondral bone cysts probably originate from the iatrogenic association of the subchondral bone marrow space with the synovial fluid, introduced either by drilling or microfracture techniques (Figure 3A), subchondral bone plate débridement, which potentially opens small vascular channels crossing into the (removed) calcified cartilage layer^32^ when preparing the defect for ACI or marrow stimulation (Figure 3B),^101^ or during OCT in cases of insufficient graft integration (Figure 3C). When performing marrow stimulation for cartilage repair, the penetrations of the subchondral bone plate generate, by definition, communications between the joint space and subchondral bone, which allows the synovial fluid to enter, serving as a possible important contributor for a subsequent subchondral bone cyst formation if these canals are preserved and not closed with an osteochondral repair tissue.^47^ In the context of OCT, the formation of subchondral bone cysts at the peripheral graft‐host interface also underscores the role of such a synovial fluid intrusion into the subchondral bone through this interface and/or eroded cartilage, resulting in subchondral bone cyst formation at an early postoperative phase.^102^, ^103^, ^104^, ^105^ Pallante‐Kichura et al found that the deterioration of the cartilaginous component of allograft OCTs seen at 1 year in adult goats was associated with subchondral cyst formation. The data suggested that a persisting lateral cartilage‐subchondral bone communication following OCT may favor fluid intrusion as a mechanism for their development, highlighting the need for further mechanistic studies to elucidate the mode of such cyst formation.^105^

Interestingly, subchondral bone cysts caused by OA in malaligned knees may regress if the mechanical overload is surgically reduced, as recently shown in a study of patients where the number of cysts located in the previously overloaded tibiofemoral compartment decreased at 5 years after unloading high tibial osteotomy.^91^ In some cases, new cysts appeared in the now overloaded lateral compartment at 5 years.^91^ These findings highlight the role of local biomechanical overload in the context of the external hydraulic theory.

### 4.2 Internal inflammatory theory

The internal inflammatory theory is based on cellular and molecular processes with upregulation of local proinflammatory factors that induce a focal area of cystic degeneration caused by an aseptic bone necrosis (as commonly seen in OCT). Local accumulation of the proinflammatory mediator prostaglandin E2 (PGE2) was identified in analyses of tissues harvested from subchondral bone cysts in horses.^68^, ^106^ Moreover, upregulation of interleukin 1 (IL‐1) and IL‐6 was detected within subchondral bone cysts.^107^ Besides, osteoclast recruiting and their activation was provoked in neonate rats when their osteoclasts were cultured in conditioned medium of the fibrous tissue and cystic fluid harvested from the center of subchondral bone cysts,^68^ which also accords to the increased number of osteoclasts and resorbed trabeculae identified at the periphery of the cystic lesions.^47^ These data might be explained by the combined effect of the inflammatory factors (e.g. PGE2, IL‐1, IL‐6, and tumor necrosis factor‐alpha), which are usually elevated in clinical cases of pathologic bone resorption.^107^, ^108^, ^109^, ^110^, ^111^, ^112^, ^113^ Placed in the context of the proposition of Woods that repetitive minor trauma to a localized area of bone results in subchondral cyst formation,^114^ it is possible that such events instigated the activation of the internal inflammatory processes.^105^

Taken together, subchondral cysts may result from the two mechanisms as described above. Posttraumatic subchondral bone cysts may develop through both mechanisms at the sites of joint injuries (e.g., fracture), possibly due to bone resorption by synovial fluid, reflected in bone marrow edema (BME),^115^ and also mechanical stress and repeated microtrauma that subsequently lead to vascular disruption, local bone necrosis, and subsequent cyst formation.^73^ As studies on OCTs have shown, channels in the lateral osteochondral graft‐host interface generated by the technique provide a communication to the joint space, which may induce subchondral bone cysts by allowing pressurized synovial fluid to enter the subchondral bone.^105^ Next, bone resorption occurs and results in the formation and expansion of a cavity that originates from the communicative canal. The host bone responds by peri‐wall bone thickening and sclerosis, which resembles the cellular and molecular processes of the internal inflammatory theory such as a local proinflammatory state with osteoclast activation, among others. It is possible that subchondral bone cysts may result from a combination of these mechanisms.

## 5 SUBCHONDRAL CYST FORMATION AFTER CLINICAL ARTICULAR CARTILAGE REPAIR

Subchondral bone cysts related to articular cartilage repair procedures are frequently observed during postoperative radiographic evaluations (Figure 1). They have been traditionally reported after marrow stimulation procedures (e.g., microfracture and subchondral drilling). Penetrations of the subchondral bone resulting from microfracture^58^ or subchondral drilling^116^ might serve as their basis. Subchondral cyst formation has also been associated with other cartilage repair procedures, among which ACI^25^, ^59^, ^60^, ^61^ and autologous or allogeneic OCT (Table 2).^62^, ^63^, ^64^ Stem cell therapy, a promising approach for cartilage repair, has not been associated with subchondral bone cyst formation based on the currently available literature and was therefore excluded from the current review. Also, as the largest number of clinical investigations on cartilage repair with long‐term follow‐ups originates from the knee, a focus is placed on this joint.

**TABLE 2:** Overview of reported subchondral bone cyst formation following the clinical use of articular cartilage repair procedures

Joint	Defect type	Index procedure	Detection method	Follow‐up (months)	Number of patients/defects	Cyst incidence per defect	Cyst characteristics				Reference
							Number	Diameter (mm)	Morphology	Location	
Knee	Chondral	Microfracture	MRI	0.8	13	0.0%	0	n.a.	n.a.	Beneath the repair site	^58^
				6	13	15.4%	2	n.a.	n.a.		
				12	13	38.5%	5	n.a.	n.a.		
				24	8	37.5%	3	n.a.	n.a.		
Knee	Chondral	1st generation ACI	MRI	155	31	38.8%	14	n.a.	n.a.	Under the lesion area	^59^
Knee	Chondral or osteochondral	1st or 2nd generation ACI	MRI	12	163	14.7%	24	Small or large; no quantification	n.a.	n.a.	^25^
Knee	Chondral; with previous failed MST	2nd generation ACI	MRI	8.4 (success); 14.4 (failure)	30 (success); 8 (failure)	6.6% (success); 37.5% (failure)	2 (success); 3 (failure)	n.a.	n.a.	n.a.	^60^
Knee.	n.a.	2nd generation ACI	Micro‐CT; histology	26.8 (before revision TKA)	10	20.0%	2	n.a.	n.a.	n.a.	^61^
Knee	Osteochondral	Allograft OCT	MRI	6	29	27.6%	8	n.a.	n.a.	Within graft or at host‐graft junction	^64^
Knee	Osteochondral	Allograft OCT with BMA	MRI	6	29	20.7%	6	n.a.	n.a.	Within graft or at host‐graft junction	^64^
Knee	Chondral or osteochondral	Allograft OCT	MRI	12	16	43.8%	7	n.a.	n.a.	Within graft or at host‐graft junction	^62^
Knee	Chondral or osteochondral	Allograft OCT	MRI	12	15	46.7%	7	n.a.	n.a.	Within graft or at host‐graft junction	^62^
Knee	Osteochondral	Allograft OCT	MRI	6	74	21.6%	16	n.a.	n.a.	Within graft or at host‐graft junction	^63^
Ankle	Osteochondral	Allograft OCT	MRI	22.3	16	62.5%	10	6.0	n.a.	Graft (9); Inferior (2); Peripheral (8)	^65^
Ankle	Osteochondral	Autograft OCT	MRI	26.3	25	40.0%	10	3.8	n.a.	Graft (1); Inferior (4); Peripheral (8)	^65^
Ankle	Osteochondral	Autograft OCT	MRI	66.3	26	76.9%	20	3.8	n.a.	Graft (7); Inferior (5); Peripheral (14)	^66^
Ankle	Osteochondral	Autograft OCT with concentrated BMA	MRI	60.8	28	46.4%	13	4.9	n.a.	Graft (4); Inferior (5); Peripheral (7)	^66^

BMA, bone marrow aspirate; micro‐CT, micro‐computed tomography; MRI, magnetic resonance imaging; MST, marrow stimulation treatment; OCT, osteochondral transplantation; TKA, total knee arthroplasty; n.a., not available.

### 5.1 Marrow stimulation

Cole and colleagues reported subchondral cysts beneath the repair tissue after microfracture of isolated full‐thickness chondral defects by MRI in 15.4% (2/13) defects at 6 months, in 38.5% (5/13) defects at 12 months, and in 37.5% (3/8) defects at 24 months postoperatively. Detailed information about cyst number, size, and morphology was not described. Noteworthy, no cysts were observed at 3 weeks postoperatively. These data suggest that subchondral bone cysts develop gradually, appearing perceptible by imaging as early as 6 months postoperatively.^58^

### 5.2 Autologous chondrocyte implantation

McCarthy et al. found subchondral bone cysts under the lesion area in 14.7% patients treated with either first‐ or second‐generation ACI at 1 year postoperatively,^25^ considerably lower than the data from previous cohorts treated with microfracture (38.5% at 1 year postoperatively) from Cole et al.^58^ Correspondingly, a recent clinical investigation from biopsies of patients undergoing total knee arthroplasty as a salvage procedure for failed second‐generation ACI with an average graft survival period of 26.8 months identified subchondral bone cyst formation within 20% of patients.^61^ In a 9‐18 years follow‐up study, subchondral cysts were reported in 38.8% knee defects treated with first‐generation ACI.^59^ Merkeley et al. identified the presence of severe BME (grade IV) as a predictive factor for graft failure (n = 8) among patients (n = 38) receiving a salvage second‐generation knee ACI for failed prior marrow stimulation. Interestingly, the incidence of subchondral cysts was not statistically significant between ACI patients without or with a prior marrow stimulation.^60^ However, in ACI patients that received a previous marrow stimulation, the incidence of cyst formation was 6.6% (2/30) in successful but 37.5% (3/8) in failed cases.^60^ Although not thoroughly addressed, these data suggest that subchondral bone cyst formation might be correlated with ACI failure in patients treated previously with marrow stimulation.

### 5.3 Osteochondral allograft transplantation

Ackermann et al compared the host‐graft integration outcomes at 1 year postoperatively after knee allograft OCT using two instrumentation sets from different companies.^62^ Outcomes were evaluated with the Osteochondral allograft MRI Scoring System, BME size, graft‐host interface distance, graft cartilage integrity, cyst size, graft contour, and effusion presence. Specifically, cysts within the graft or at the host‐graft junction were observed in 43.8% (7/16) and 46.7% (7/15) cases without a statistically significant difference between the two instrumentation sets. These data indicate a considerable incidence of subchondral bone cyst following allograft OCT at 1 year postoperatively that is well within the range reported for microfracture^58^ and not affected by the choice of instrumentation.

In patients with focal knee osteochondral defects, cysts within the graft or at the host‐graft junction were observed at 6 months postoperatively in 27.6% (8/29) and 20.7% (6/29) of patients treated with allograft OCT without or with unconcentrated bone marrow aspirate (BMA) without a statistically significant difference between the groups.^64^

### 5.4 Subchondral bone cyst formation in other joints

Of special note, subchondral cyst formation has also been associated with femoroacetabular impingement^117^ and following cartilage surgeries in other joints, especially the ankle. By morphological analysis, subchondral talar cysts are either of an irregular or round shape. ^118^ Cysts with an opening through the subchondral bone plate into the joint space can sometimes be identified. The presence of a sclerotic rim is reflected in the higher peri‐cyst bone volume fraction than in the normal subarticular spongiosa.^119^ Allograft^65^ or autograft^66^ OCTs have been associated with the occurrence of cysts in the ankle joint. Most cysts are located peripheral to or within the grafts. Comparing the clinical and radiographic outcomes of autograft and allograft OCT to treat talar osteochondral defects, Shimozono et al. identified a statistically nonsignificant trend of more subchondral bone cyst formation in 62.5% of cases (10/16) treated with allograft OCT (autograft OCT: 40.0%, 10/25). ^65^ Interestingly, autograft OCT also yielded a significantly improved ankle function and superior Magnetic Resonance Observation of Cartilage Repair Tissue (MOCART) score compared with allograft OCT at about 2 years postoperatively, in good agreement with the significantly higher rate of clinical failures following allograft (25%, 4/16) compared with autograft OCT (0%, 0/25).^65^

Shimozono et al. also compared the postoperative incidence of cysts in ankle autograft OCT without or with concentrated BMA with a 60 months’ follow‐up. ^66^ The cyst incidence was significantly lower in autograft OCT with concentrated BMA (46.4%, 13/28) than in OCT without concentrated BMA (76.9%, 20/26). However, the cyst size and location were comparable between both groups. These data show a long‐term favorable inhibitory effectiveness of concentrated BMA against postoperative cyst formation after ankle autograft OCT, which is opposed to the short‐term data from Ackermann et al after knee allograft OCT.^64^ These findings suggest that outcomes reported for the ankle joint may not be straightforwardly translated to the knee joint. Also, more investigations are needed to elucidate the possible varied efficacy of combinations with OCT (allograft or autograft) augmented with BMA (unconcentrated or concentrated) in different joints.

### 5.5 Association of postoperative subchondral cyst formation and clinical outcomes of articular cartilage repair

Although subchondral bone cysts represent an early postoperative sign associated with many articular cartilage repair procedures, a possible association between them and inferior clinical outcomes has not been well established in either the knee or the ankle joint.^51^ An association between BME and subchondral bone cysts was already confirmed in the context of OA. Carrino et al showed that cysts always arose from regions of BME‐like signals in knee OA patients (n = 32) after a mean of 17.5 months (range 2.1‐40.1 months). BME were detected in 68 subarticular areas and 23 cysts. Interestingly, increases in size were noted for 25% of the BME and 26.1% of the cysts, 25.0% of BME and 4.4% of cysts decreased, while 21.7% of BME and 1.5% of cysts were unchanged (23.5% of BME were new, 16.2% were resolved). The BME signal size always changed with the cyst development: it increased in 54.5%, decreased in 18.1%, and resolved in 27.2% of cases. Of note, a change in cyst size was constantly accompanied by a change in edema‐like signal size. Moreover, an abnormality of the adjacent articular cartilage was identified for 87.0% of the cysts by MRI.^120^ Further evidence supports the association of BME and subchondral bone cysts, and the BME signal on MRI has been also statistically linked with degenerative articular cartilage loss^121^ or cartilage defects.^121^ Arthroscopic grades of knee articular cartilage defects are positively associated with the prevalence, depth, and cross‐sectional area of subchondral BME on MRI.^122^ Also, BME grading has been positively correlated to the presence of knee pain and stiffness, radiographic severity, and the increased rate of OA progression.^123^, ^124^, ^125^ These findings attest to the strong relationships between the BMA and the occurrence of subchondral cysts and suggest an influence on clinical outcomes in the specific context of OA.

However, such a relationship may not be directly inferred to the different settings of the repair of focal cartilage defects in the knee. Currently available cross‐sectional^25^, ^59^, ^61^, ^66^ or cohort^58^, ^65^ studies do not allow identifying a causal relationship between the occurrence of subchondral bone cysts and clinical or radiographic outcomes of articular cartilage repair. For instance, ACI graft failure has been associated with BME, as the rate in patients with severe BME (83.7%) was significantly higher than in patients without severe BME (6.5%) at 60 months postoperatively.^60^ In contrast, Vasiliadis et al identified a higher risk for subchondral bone cysts after ACI at 3 years postoperatively, which was not associated with the occurrence of BME but with increasing patient age.^59^ These data underscore the pathophysiological and clinical different phenotypes of OA and focal (non‐OA) cartilage defects, which complicate a simple transfer of the rather large evidence gained from the OA field into the context of cartilage repair. They call for more individual investigations into the natural course of cartilage defects and their repair, with a special attention to OA development since these defects are possible triggers to develop secondary OA.^126^, ^127^ However, the strong evidence identifying BME as a risk factor for structural progression of knee OA, together with the proposition that BME represents a ‘‘pre‐cyst’’ sign^120^ (although not every area of BME may give rise to a cyst)^120^ warrants clinical alertness to prolonged symptomatic cases following articular cartilage repair, thus necessitating the need for staged MRI to rule out the possibility of BME and, if present, its appropriate treatment to avoid a possible conversion of such a BME into a subchondral bone cyst.

Controversial data have been accumulated for the ankle joint.^66^, ^128^, ^129^ Evidence of cystic changes was identified in 65.8% patients using MRI after autograft OCT for talar osteochondral defects at a short‐term follow‐up of 15 months postoperatively.^129^ Interestingly, subchondral bone cyst formation was neither correlated with cartilage integrity nor patient‐reported outcomes.^129^ In another study, postoperative cyst formation did not affect clinical outcomes of talar autograft OCT for osteochondral defects. Subchondral cysts were identified via MRI in 64.8% of patients at 15 months (range, 2‐54) postoperatively.^128^ Patients with postoperative cysts were significantly older than those without cysts (mean age, 42.7 vs 32.7 years). Among the patients with a cyst, the subchondral bone plate was significantly more involved in old patients (57.3 vs 36.7 years). Interestingly, no other variables associated with cyst formation achieved statistical significance. Patients without postoperative cysts were characterized by lower preoperative Short Form‐12 (SF‐12) and Foot and Ankle Outcome Score (FAOS) and significantly more postoperative improvements in both scores than patients that developed cysts. However, a long‐term study of autograft OCT without or with concentrated bone marrow for talar osteochondral defects reported no significant differences of the postoperative SF‐12 and FAOS between patients without or with cysts at a follow‐up at 5 years postoperatively.^66^

## 6 SUBCHONDRAL CYST FORMATION IN PRECLINICAL CARTILAGE REPAIR

Subchondral bone cyst formation following articular cartilage repair in preclinical models has been regularly recognized as a common postoperative phenomenon.^46^, ^47^, ^48^, ^56^, ^57^ Such preclinical models offer the elegant possibility of performing ex vivo analyses of the microstructure of the subchondral bone a high resolution using micro‐computed tomography (micro‐CT), allowing to depict subchondral bone cysts at a magnitude of detail that is difficult to obtain in clinical settings.^46^, ^47^, ^57^, ^116^, ^130^ The prevalence of subchondral bone cyst formation following cartilage repair can be as high as 92.0% in sheep at 6 months postoperatively.^47^ Although subchondral bone cyst formation appears to be species and procedure specific, detailed attention to this important issue appears to be warranted (Table 3).^55^, ^56^

**TABLE 3:** Overview of reported subchondral bone cyst formation following the preclinical use of articular cartilage repair procedures

Preclinicalmodel	Joint	Defect type	Index procedure	Detection method	Follow‐up (months)	Number of animal	Incidence per defect	Reference
Horse	Knee	Osteochondral	Spontaneous repair	Histomorphometry	12	10	n.a.	^53^
Horse	Knee	Osteochondral	Spontaneous repair	Xeroradiography	4	3	n.a.	^54^
Sheep	Knee	Chondral	Microfracture	Histomorphometry; micro‐CT	3.3; 6.5	12	33.3% (3.3 m); 91.7% (6.5 m)*	^47^
Sheep	Knee	Chondral	Microfracture	Histomorphometry; micro‐CT	3	8	25%	^46^
Sheep	Knee	Chondral	Microfracture	Histomorphometry	6	6	83%	^56^
Horse	Knee	Chondral	Microfracture	Histomorphometry	4	5	0%	^55^
Horse	Knee	Chondral	Microfracture	Histomorphometry	12	5	10%	^55^
Sheep	Knee	Chondral	Microfracture	Histomorphometry	6	8	63%	^56^
Rabbit	Knee	Chondral	Drilling	Histomorphometry; micro‐CT	3	8	41%	^46^
Sheep	Knee	Chondral	Drilling	Histomorphometry; micro‐CT	6	19	63%	^57^
Sheep	Knee	Chondral	AMIC	Histomorphometry; micro‐CT	3.3; 6.5	12	3.3 m: 50% (3.3 m); 91.7% (6.5 m)*	^47^

AMIC, Autologous matrix‐induced chondrogenesis; micro‐CT, micro‐computed tomography; n.a., not available. *The mean incidence of subchondral bone cyst formation was 91.7% in samples treated by either microfracture or AMIC at 6.5 month postoperatively, however, no detailed information regarding cyst formation rate for each procedure was separately provided.^47.^

### 6.1 Spontaneous cartilage repair

Subchondral cyst formation during the spontaneous repair of osteochondral defects may also be location dependent. In a minipig model, more frequent subchondral bone cyst formation was seen in the medial femoral condyle compared with the medial patellar groove at 12 months postoperatively.^131^ Other studies of spontaneous cartilage repair applying either histomorphometry^53^ or xeroradiography^54^ did not address the issue of subchondral bone cyst formation.

### 6.2 Microfracture and augmented procedures

The incidence of subchondral bone cysts after microfracture for knee chondral defects was 25% at 3 months postoperatively in rabbits.^46^ However, several studies in the ovine model revealed a much higher incidence of 83‐92% at 6 months postoperatively when analyzed with micro‐CT.^47^, ^56^, ^130^ Communication through the microfracture holes between the intraarticular space and the subchondral bone cysts persevered for up to 6 months postoperatively,^47^ highlighting the potential role of the surgical penetrations of the subchondral bone with the microfracture instruments as a possible factor that may essentially be involved in subchondral bone cyst formation over time.^102^ Prevalence of subchondral bone cyst formation was 50% at 3 months postoperatively and 92% at 6 months postoperatively following autologous matrix‐induced chondrogenesis (AMIC) in sheep,^47^ which was comparable to the outcome of microfracture alone at both time points (33.3% and 91.7%, respectively). These data suggest that utilizing an additional bioresorbable membrane scaffold may not reduce the early formation of subchondral bone cysts after microfracture. Also, these early subchondral bone alterations might partly explain the comparable clinical and radiographic outcomes between AMIC and microfracture for knee chondral defects at 5 years postoperatively.^132^, ^133^

### 6.3 Subchondral drilling

The occurrence of subchondral bone cysts was as high as 41% in a rabbit model at 3 months postoperatively after subchondral drilling for chondral defects of the knee.^46^ Orth et al reported that subchondral drilling for full‐thickness chondral defects in the medial femoral condyle of sheep led to the formation of subchondral bone cysts in 63% of defects at 6 months postoperatively.^57^ These bone cysts always originated from the canals generated during the drilling with Kirschner wires. Of note, multiple cysts can concurrently originate from one single defect, and the cyst dimension may also largely exceed the original defect area.

### 6.4 Autologous chondrocyte implantation

Subchondral bone cyst formation has only rarely been reported in preclinical models of autologous chondrocyte implantation (ACI). A chondrocyte suspension was applied to cartilage defects in a goat model, sealed by a periosteal flap or a collagen membrane, and evaluated after 10 weeks in vivo. If the treated defects were not filled with a repair tissue and if the calcified layer and subchondral bone were damaged at this early time point, bone cracks and subchondral bone cysts below the defect were revealed by histological analysis. Such subchondral bone cyst formation associated with graft failure was limited, although no details on their incidence were reported.^134^

## 7 CLINICAL IMPLICATIONS AND OUTLOOK

Due to its high incidence (38.5% in microfracture^58^; 38.8% in ACI^59^; 38.9% in allograft OCT^62^; 62.5% in allograft OCT^65^; 76.9% in autograft OCT^66^) and lasting presence (over 12 years reported for ACI^59^), subchondral bone cyst formation following articular cartilage repair merits serious attention. Its appearance as early as 6 months postoperatively in over 15% of patients treated with microfracture^58^ and 20% of patients treated with OCT^63^, ^64^ highlights the clinical importance.^57^ The currently recommended timeframe for touchdown weight‐bearing within the first 6‐8 postoperative weeks and free full weight‐bearing thereafter, therefore, needs to be respected to constrain early subchondral bone changes,^5^, ^135^ considering the fact that the rather small bone defects resulting from marrow stimulation will be closed after such a period, but possibly not if earlier weight‐bearing pushes the synovial fluid through the soft repair tissue into the residual (subchondral) canals from the marrow stimulation or, in the case of OCT, into the nonintegrated interface between the graft and the adjacent normal osteochondral unit. This, in turn, might possibly lead to the bone resorption and/or remodeling seen on MRI as BME, followed by subchondral bone cyst formation that ultimately weakens the osteochondral unit that leads to its deterioration over time.

Besides, these data also prompt more clinical observations and radiographic follow‐ups (e.g., MRI or cone‐beam CT^136^) during the early postoperative phase to identify premature subchondral bone changes for the possible optimization and individualization of the rehabilitation program. As already stated, MRI evaluations over time (e.g. in 6‐week intervals) may be indicated in cases of prolonged pain following the different cartilage repair procedures to rule out BME and/or subchondral bone cysts. Likewise, a long‐term follow‐up of postoperative subchondral bone cysts appears mandatory for many of the cartilage repair techniques.

The substantial inconsistency in the terminology used to describe entities of subchondral bone changes has already been recognized as a frequent disconnect between the used nomenclature and the actual morphological change.^32^, ^51^, ^52^ Standardization of analyses and outcome reporting of postoperative subchondral bone changes, possibly with an established algorithm will assist investigators to report salient characteristics of subchondral bone changes and to improve the transparency and comparability of data from studies regarding articular cartilage repair.^52^

A number of other specific issues and possible research questions are worthy to be addressed to further optimize cartilage repair in a clinical setting. First, a better understanding of the mechanisms of subchondral bone cyst development will improve surgical treatment and postoperative rehabilitation and prevent further cyst formation. Second, continuous updating and augmenting the currently available techniques are necessary to reduce or even avoid these deleterious subchondral bone changes. For example, concentrated BMA‐enhanced autograft OCT was shown to decrease subchondral cyst formation rate for talar osteochondral defects,^66^ however no information is available about the knee joint. Similarly, it will be interesting to see if the additional coverage of microfractured cartilage defects with biomaterials (e.g., membrane scaffolds) might result in a lower incidence of subchondral cyst formation and possibly ensure better long‐term outcomes compared with the traditional marrow stimulation technique. Third, for ACI, a possible correlation of subchondral bone cyst formation and ACI failure remains to be investigated. Lastly, for an already established subchondral bone cyst, salvage managements (e.g., curettage and autologous cancellous bone grafting) might be beneficial to guarantee the long‐term success of the index cartilage repair procedure.^137^

## 8 CONCLUSION

Subchondral bone cysts are one of the most widely reported subchondral bone changes associated with the repair of focal articular cartilage defects. More investigations into their mechanisms of development and both clinical and radiographic follow‐up in the context of specific cartilage repair procedures will enhance our understanding of the important relationships between the occurrence of postoperative subchondral cysts and clinical outcomes in cartilage repair.

## AUTHOR CONTRIBUTIONS

Henning Madry provided the conception and designing. Liang Gao, Magali Cucchiarini, and Henning Madry contributed to literature searching and wrote the manuscript. All authors read and approved the final manuscript.

### CONFLICT OF INTEREST

The authors declare that they have no conflict of interest.
